# Working hard for recovery: mitotic kinases in the DNA damage checkpoint

**DOI:** 10.1186/2045-3701-3-20

**Published:** 2013-04-23

**Authors:** Aimin Peng

**Affiliations:** 1 Department of Oral Biology, College of Dentistry, University of Nebraska Medical Center, Lincoln, NE 68583, USA

**Keywords:** DNA damage checkpoint, Recovery, Mitotic kinases, Cdk, Plk1, Aurora, Gwl

## Abstract

Cell division in mitosis is tightly regulated via a group of protein kinases. Activation of these mitotic kinases is inhibited by the DNA damage checkpoint that arrests the cell cycle in interphase and prevents mitotic entry. Interestingly, it has been shown that the DNA damage checkpoint is feedback regulated by several mitotic kinases. These kinases are reactivated from checkpoint arrest to deactivate the checkpoint and restart cell cycle progression, thereby allowing the cell to recover from the DNA damage checkpoint. The emerging role of mitotic kinases in the DNA damage pathway provides important insights into cancer progression and treatment.

## Introduction

DNA damage is frequently induced by radiation, genotoxic chemicals, and metabolic byproducts, posing enormous threats on genomic integrity. Upon DNA damage, the cell must activate the DNA damage checkpoint to halt cell cycle progression until DNA repair is completed [1,2]. It has been shown that the DNA damage checkpoint prevents activation of cyclin-dependent kinases (Cdks) and several other essential mitotic kinases, such as Polo-like kinase 1 (Plk1), Aurora A and Greatwall (Gwl). Because these mitotic kinases are inhibited by DNA damage, a prevailing assumption has been that they remain inactive until the checkpoint signal is turned off. However, recent studies demonstrated a surprising role of these mitotic kinases in deactivating the checkpoint and promoting cell cycle reentry in interphase, a process called checkpoint recovery. These studies thereby suggested a reciprocal regulation of the DNA damage checkpoint by mitotic kinases. For example, Plk1 phosphorylates several checkpoint factors, leading to their deactivation, and has been therefore recognized as a key player of checkpoint recovery [3,4]. By comparison, the involvement of other mitotic kinases in checkpoint recovery has been implicated but less understood. This review will summarize recent studies in the field, and discuss how these findings may impact our understanding of cancer progression and therapy.

### Activation of the DNA damage checkpoint

Various forms of DNA damage can be induced in cells by endogenous or exogenous agents. An important part of the cellular response to DNA damage is to stop cell cycle progression when DNA damage is sensed. This mechanism, termed the DNA damage checkpoint, is believed to facilitate DNA repair and prevent genomic instability [1]. The essential role of the checkpoint is highlighted by the fact that genetic mutations of the pathway are often implicated in human diseases. In particular, DNA damage checkpoint genes, including *ATM, TP53, BRCA1, CHEK2, NBS1, MRE11*, were found frequently mutated in familial cancer patients with various penetrance, indicating a critical tumor suppression role of the pathway [2,5].

Extensive research efforts were dedicated to revealing the molecular network of the DNA damage checkpoint. It is now well-established that a key event following DNA damage is activation of ATM and ATR, two phosphatidylinositol 3-kinase-related kinases (PIKKs) that are widely regarded as sensor kinases. Upon activation, ATM and ATR phosphorylate a large number of substrate proteins to regulate various downstream pathways, including DNA repair, checkpoint activation, and cell death [6]. Activation of the DNA damage checkpoint is mediated by two checkpoint kinases, Chk1 and Chk2. These kinases are phosphorylated and activated by ATR and ATM, and in turn phosphorylate substrates such as Cdc25 and p53 to control cell cycle progression [1]. Cdc25 functions as an essential activator of Cdks by dephosphorylating Cdks at inhibitory sites [7]; p53 activates transcription of p21 which then inhibits Cdk activation [1]. Thus, the current model of the DNA damage checkpoint is centered at inhibitory regulation of Cdks, such as Cdk1/Cyclin B, the principal mitotic kinase whose inhibition upon activation of the G2/M DNA damage checkpoint would effectively block mitotic entry.

In addition to Cdk1, there are a number of other kinases whose activities also oscillate during the cell cycle and peak in mitosis. Many of these kinases are believed to play important roles in mitotic entry, progression, and exit. For example, polo-like kinase 1 (Plk1) is a well-studied mitotic kinase whose inactivation leads to multiple mitotic defects and cell death. Plk1 contains a kinase domain and a characteristic polo-box domain that is involved in determination of subcellular localization and substrate selectivity. Upon activation, Plk1 phosphorylates a wide range of substrates, including Cdc25, Wee1, Emi2, Bora, etc., involved in various aspects of mitosis [3,4]. Interestingly, recent studies indicated that Plk1 is activated by another well-characterized mitotic kinase named Aurora A, which phosphorylates Plk1 at its T-loop activating site [8,9]. Aurora A phosphorylates and regulates numerous mitotic substrates, whereas the function of Aurora A is mediated by various activators/co-factors, including Bora, Tpx2, Ajuba, Hef1, etc. [3,10]. Aurora A belongs to the Aurora family of kinases along with Aurora B and C. Despite similarities in structure and substrate recognition, Aurora B and Aurora A exhibit distinct patterns of subcellular localization and protein interaction [11,12]. Moreover, recent studies revealed that mitotic entry and maintenance are dependent on a relatively less-studied kinase named Greatwall (Gwl, as known as Mastl: microtubule-associated serine/threonine kinase like). Gwl phosphorylates Ensa and Arpp-19, two related factors that, upon phosphorylation, specifically bind and inhibit a protein phosphatase complex named PP2A/B55δ. Inhibition of this phosphatase complex prevents it from dephosphorylating Cdk1 substrates, and is therefore a prerequisite for mitotic entry and maintenance [13-16]. Readers are referred to several excellent reviews for detailed functions and regulatory mechanisms of these mitotic kinases [3,4,10-17].

**Figure 1:**
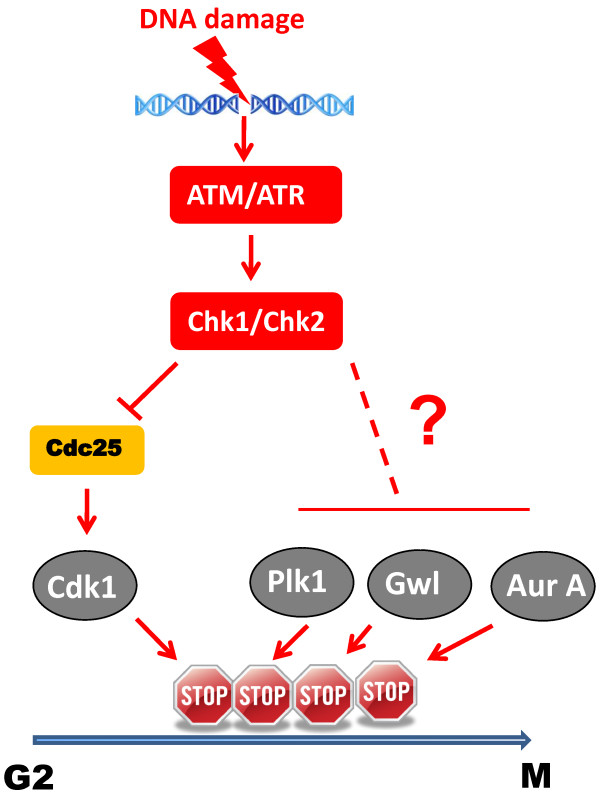
**A multi-brake model of the G2/M DNA damage checkpoint. **Activation of the DNA damage checkpoint and maintenance of cell cycle arrest require not only inhibition of Cdk1, but also independent targeting of Plk1, Aurora A and Gwl. Detailed mechanisms through which the DNA damage checkpoint suppresses Plk1, Aurora A and Gwl kinases remain to be clarified (as denoted by the question mark).

Interestingly, emerging evidence indicated that many of these non-Cdk mitotic kinases are also targeted by the G2/M DNA damage checkpoint, presumably to reinforce the G2 arrest resulted from Cdk1 inhibition. It has been shown that Plk1 activation is inhibited by DNA damage [18,19]. Importantly, expression of a constitutively active form of Plk1 overrides the G2/M DNA damage checkpoint, indicating an essential involvement of Plk1 inhibition in checkpoint activation [18]. It is still unclear how DNA damage leads to Plk1 inhibition during G2/M transition. PP2A-mediated dephosphorylation has been suggested as a potent mechanism of Plk1 deactivation because Plk1 activation is triggered by phosphorylation at its activating site. In mitosis, some studies [20,21], although not others [22], found that DNA damage leads to Plk1 dephosphorylation. Interestingly, Aurora A is also inhibited by DNA damage in a manner that is independent of Cdk inhibition [23]. Aurora A inhibition by DNA damage requires Chk1-dependent signaling, and is somehow dependent on phosphorylation of Ser-342 of Aurora A. Overexpression of wild-type or Ser-342 to Ala-mutant Aurora A in DNA damage-treated cells led to checkpoint bypass and mitotic entry, suggesting a functional significance of Aurora A inhibition in the DNA damage checkpoint [23]. Finally, our recent study in *Xenopus* egg extracts discovered inhibition of Gwl kinase by DNA damage: pre-activated Gwl is more efficiently deactivated in extracts supplemented with DNA damage compared to the control interphase extract; such inhibition is sensitive to caffeine, an inhibitor of ATM/ATR [24]. Collectively, these recent studies suggest a “multi-brake” model of the G2/M DNA damage checkpoint, in which the checkpoint targets not only Cdk1, but also several other mitotic kinases, and like that of Cdk1, inhibition of these non-Cdk kinases is essential for activation and maintenance of the DNA damage checkpoint (Figure 1).

### Deactivation of the DNA damage checkpoint—checkpoint recovery

Activation of the DNA damage checkpoint allows the cell to successfully repair DNA damage. Upon completion of DNA repair, the cell deactivates the checkpoint and resumes cell cycle progression, which process is termed checkpoint recovery. Compared to activation of the DNA damage checkpoint, checkpoint recovery is much less understood, and was only recently appreciated as an active and regulated process [25,26]. Giving that the checkpoint is activated largely through protein phosphorylation and kinase cascades, the emerging role of protein phosphatases in checkpoint recovery is not surprising [27]. In particular, PP2Cδ, also known as Wip1 (wild-type p53-induced phosphatase 1) has been shown to antagonize activation of multiple components of the stress and DNA damage response pathways. Expression of Wip1 is enhanced hours after DNA damage in a p53-dependent manner; Wip1 then dephosphorylates and deactivates ATM, γ-H2AX, Chk1, Chk2 and other DNA damage checkpoint factors, thereby promoting checkpoint recovery [28,29]. Interestingly, several mitotic kinases, including Plk1, Aurora A, Cdk1 and Gwl, emerged as another class of factors required for checkpoint recovery. The involvement of these kinases in checkpoint recovery is further discussed below:

*Plk1*. The role of Plk1 as an essential regulator of checkpoint recovery from DNA damage-induced G2 arrest was first reported by van Vugt et al. [30,31]. Interestingly, this study showed that down-regulation of Plk1, which did not block mitotic entry in unperturbed cell cycle, significantly delayed mitotic reentry after DNA damage [30]. Such a differential requirement of Plk1 for checkpoint recovery and for normal cell cycle progression suggests specific functions of Plk1 in checkpoint recovery. The checkpoint recovery deficiency in Plk1-suppressed cells can be rescued by depletion of Wee1, a tyrosine kinase that inhibits Cdk activation, thereby suggesting Wee1 as a crucial downstream factor of Plk1 in promoting checkpoint recovery [30]. More recently, the role of Plk1 in checkpoint recovery was further revealed through characterization of Claspin as a direct target of Plk1 during the recovery process. Claspin functions as a mediator protein that allows ATR to phosphorylate and activate Chk1 [32]. Yoo et al. first discovered in *Xenopus* egg extracts that Plk1 phosphorylates Claspin after prolonged interphase arrest in aphidicolin-treated extracts, leading to release of Claspin from chromatin and checkpoint inactivation [33]. While this study was conducted within the context of checkpoint adaptation, a term specifically referring to cell recovery despite presence of DNA damage, from replication checkpoint, this role of Plk1 in checkpoint deactivation through Claspin regulation may also account for DNA damage checkpoint recovery. Indeed, a similar mode of regulation was then reported in human cells with independent studies from several laboratories confirming Plk1-dependent phosphorylation of Claspin as a crucial event of checkpoint recovery. Interestingly, these elegant studies showed that Plk1-dependent phosphorylation directs Claspin to Skp/Cullin/F-box-β-Transducin repeat containing protein (SCF-βTrCP)-mediated ubiquitination and proteolysis [34-36]. More recently, it has been shown that Plk1 phosphorylates Chk2 and 53BP1, a checkpoint protein that mediates ATM-dependent phosphorylation of downstream factors. These phosphorylation events disrupt the function of 53BP1 and Chk2 in checkpoint signaling [37]. Collectively, the studies summarized above revealed an essential role of Plk1 in checkpoint recovery by directly targeting multiple DNA damage checkpoint factors, and thereby allowing checkpoint-deactivation and cell cycle reentry.

*Aurora A*. It has been shown that activation of Plk1 is dependent on Aurora A during both a normal mitosis and checkpoint recovery [8,9]. When complexed to a cofactor named Bora, Aurora A phosphorylates Plk1 at Thr-210, the T-loop activation site of Plk1. Both Aurora A and Bora are required for checkpoint recovery and mitotic reentry after DNA damage, and not so surprisingly, the requirement of Aurora A for checkpoint recovery is overcome by expression of a constitutively active form of Plk1, indicating that Aurora A promotes checkpoint recovery largely through Plk1 [8].

*Cdk*. Cdks are central regulators of the cell cycle. Cdk1, in particular, is essential for the cell to enter mitosis, and must be inhibited during activation of the G2 DNA damage checkpoint. The notion that Cdk1 activation is suppressed by the DNA damage checkpoint seemingly contradicts with any possible role of Cdk1 in the DNA damage response. However, Cdk1 has been shown to function in various aspects of DNA repair and the DNA damage checkpoint [38,39]. A crucial role of Cdk1 in DNA repair, especially homologous recombination, has been shown in yeast and human cells [40-45]. Cdk1 activity was found necessary for proper DNA end resection, a critical step of homologous recombination; and consistently, a number of studies revealed that Cdk1-dependent phosphorylation of CtIP and other repair proteins mediates their functions in DNA end resection [44-49]. In addition to DNA repair, Ira el al. also found in yeast that Cdk1 is required for double strand break-induced checkpoint activation [40]. This function of Cdk1 is likely to be conserved in human cells [50], and may be attributed to both Cdk1-mediated DNA end resection that is required for ATR activation [51], and direct phosphorylation of Chk1 that is required for efficient activation of Chk1 [52,53]. The role of Cdk1 in regulating DNA repair and initiating checkpoint activation may reflect its molecular actions before DNA damage occurs or at least before the checkpoint is fully established. However, Cdk1 has also been shown to function in checkpoint recovery, a process that is, ironically, required for reactivation of Cdk1 and mitotic reentry. In fission yeast, Cdk1 phosphorylates Crb2, the homolog of Rad9 in budding yeast and 53BP1/Mdc1/Brca1 in human; this phosphorylation occurs during the late stage of the DNA damage response and is required for cell cycle reentry after DNA damage [54]. Conserved to Cdk1-dependent phosphorylation of Crb2 in fission yeast, human 53BP1 is phosphorylated by Cdk1 in mitosis. This phosphorylation by Cdk1 then primes for further phosphorylation of 53BP1 by Plk1, and eventually leading to deactivation of 53BP1 in the checkpoint signaling pathway [37]. Similarly, Mdc1 has also been suggested as a substrate of Cdk1, and Cdk1-dependent regulation disrupts Mdc1 and γ-H2AX interaction, presumably to avoid checkpoint activation during mitosis [55]. As these studies were focused on mitotic regulation of the DNA damage checkpoint, additional evidence is required to link these Cdk1-mediated phosphorylation events with checkpoint recovery in interphase. Interestingly, a different mode of mechanism by which Cdk activity promotes checkpoint recovery has been revealed [56,57]. Alvarez-Fernandez et al. showed that recovery from the G2 DNA damage checkpoint requires Cdk-dependent phosphorylation and activation of FoxM1, which at G2 controls transcription of Cyclin A, Cyclin B, Plk1 and other cell cycle genes [56]. These authors suggested an interesting model in which Cdk activity is preserved at a low level during the DNA damage response, and such residual Cdk activity is required for maintaining the competency of the cell for checkpoint recovery. Although the authors found no apparent physiological importance of this residual activity for an unperturbed cell cycle, its inhibition led to failure of checkpoint recovery. The Cdk activity was shown to be dependent on Cyclin A rather than Cyclin B [56]. Clearly, further characterization of this Cdk activity, especially how it is protected from the DNA damage checkpoint, and its detailed molecular actions, will substantially improve our understanding of checkpoint recovery.

*Gwl*. First identified in *Drosophila*, Greatwall (Gwl) has been extensively characterized in *Xenopus* and human cells as an essential mitotic kinase [13,16]. Interestingly, we recently discovered an involvement of Gwl in checkpoint recovery [24,58,59]. Depletion of Gwl from *Xenopus* egg extracts impaired checkpoint recovery, as judged by persistent activation of checkpoint proteins and delayed reactivation of Cdk1 after removal of DNA damage. Conversely, overexpression of wild-type, but not kinase-dead, Gwl suppressed checkpoint signaling in response to DNA damage [24]. While these results established Gwl as an essential regulator of checkpoint recovery, it remains unclear how Gwl functions in the recovery process. For example, does Gwl regulate the DNA damage checkpoint through Ensa and Arpp-19, currently the only known substrates of Gwl, or alternatively, could Gwl target other factors involved in the DNA damage checkpoint pathway?

### Future perspectives

Though checkpoint recovery is still understood to a much less extent compared to checkpoint activation, a great deal of knowledge about this process has been learned through revealing the involvement of mitotic kinases. However, with the emerging role of multiple mitotic kinases in checkpoint recovery arise many intriguing questions, in particular, their regulation, mutual relationship, and implications to cancer. Continued research efforts are required to address these key questions in the near future.

#### How are mitotic kinases reactivated during checkpoint recovery

**Figure 2:**
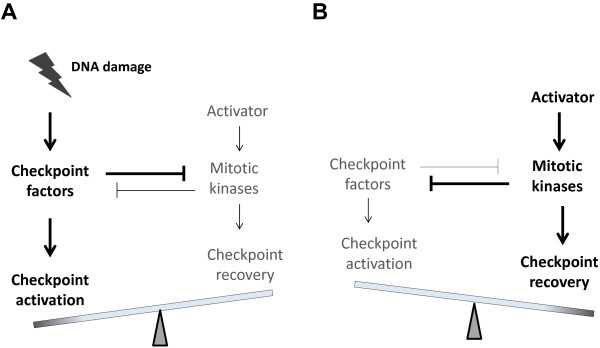
**A balance model of checkpoint recovery. **The choice between checkpoint activation (**A**) and recovery (**B**) is controlled by reciprocal regulation of checkpoint factors and mitotic kinases. Establishment of the DNA damage checkpoint inhibits full-activation of mitotic kinases, which however, retain residual levels of activities. Checkpoint recovery can be initiated as the balance between the checkpoint machinery and mitotic kinases shifts: reduced checkpoint signaling leads to increased activities of mitotic kinases, which then further suppress checkpoint signaling through feedback regulation.

Current understanding of the role of mitotic kinases in checkpoint recovery can be summarized as: 1) these kinases are inhibited by the DNA damage checkpoint to allow checkpoint activation and prevent mitosis; and 2) reactivation of these kinases deactivates checkpoint signaling and leads to cell recovery. These two lines of evidence are seemingly contradictory and disconnected by a major gap-in-knowledge: How do these kinases reactivate from checkpoint arrest? In principle, reactivation of these kinases before checkpoint deactivation may suggest two possibilities: 1) an internal timer-like mechanism evokes activation of these kinases from checkpoint arrest even with unrepaired DNA damage and persistent checkpoint signaling; 2) the checkpoint does not fully abrogate activation of these mitotic kinases, which maintain a residual level of activities as a result of both activating and suppressing signals. During checkpoint recovery, the suppressing signal reduces owing to progression of DNA repair, and thereby shifting the balance toward further activation of these kinases (Figure 2).

The core of the timer model lies in that checkpoint recovery can be committed spontaneously after prolonged checkpoint arrest with or without completion of DNA repair, which notion was possibly reflected in previous studies that revealed a transient nature of the G2/M damage checkpoint [60,61]. Moreover, spontaneous checkpoint recovery without completion of DNA repair is consistent with the checkpoint adaptation phenomenon characterized in yeast, *Xenopus*, and mammalian cells [25,26]. In particular, the G2/M DNA damage checkpoint in mammalian cells has been described as “imperfect” for allowing cell cycle reentry without completion of DNA repair [62,63].

The balance model is supported by the recent observation of residual activities of mitotic kinases during checkpoint arrest. In theory these activities can be quickly elevated with the weakening of checkpoint signals, leading to further deactivation of checkpoint signals, and eventually mitotic reentry (Figure 2). An elegant example of such theory was presented in the previous study that discovered residual Cdk activity during DNA damage-induced G2 arrest and demonstrated an essential role of this activity in conferring cell competency for checkpoint recovery [56]. Moreover, our previous study in *Xenopus* egg extracts also noted a residual activity of Gwl kinase in interphase egg extracts treated with DNA damage [24].

Given the important role of Plk1 and other mitotic kinases in checkpoint recovery, it is plausible that reactivation of these kinases from checkpoint arrest is a critical point of checkpoint recovery. The timer model and balance model can both account for reactivation of these kinases as such process is likely to involve coordinated actions of distinct mechanisms. Future delineation of these molecular mechanisms should substantially advance our understanding of how the cell transits from the state of checkpoint activation to recovery.

#### How is checkpoint recovery related to cancer progression and treatment

**Figure 3:**
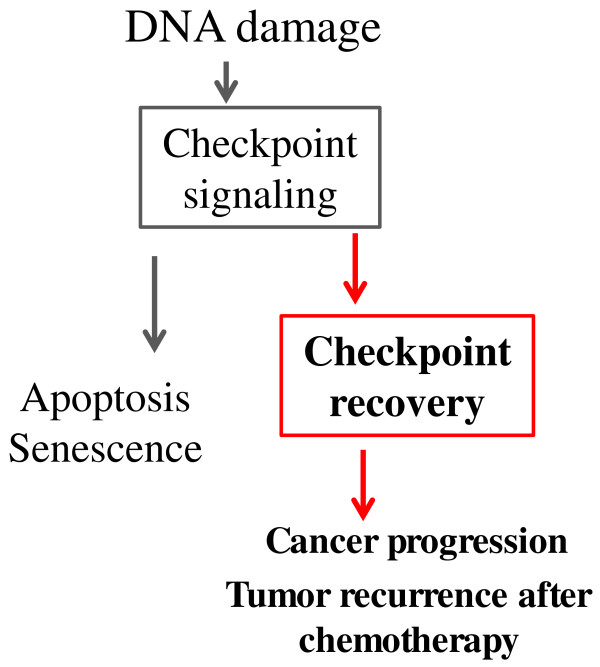
**Checkpoint recovery allows cancer cells to bypass the DNA damage checkpoint during cancer progression and therapy. **Therefore, checkpoint recovery is of great interests to better understanding tumorigenesis, improving cancer diagnosis, and developing future therapeutics.

The critical role of the DNA damage checkpoint pathway in cancer progression has been firmly established, owing to the fact that germline mutations of checkpoint genes lead to genomic instability syndromes characterized by developmental defects, aging phenotypes, and particularly, predisposition to cancers. For example, ataxia-telangiectasia (AT) is an autosomal recessive disorder resulting from loss of ATM, and associated with lymphoid malignancy along with other phenotypes [6]. Similarly, a variety of disorders have been attributed to mutations in other checkpoint factors, such as NBS1 in Nijmegen breakage syndrome, Mre11 in AT-like disorder, ATR in Seckel syndrome, Chk2 and p53 in Li-Fraumeni syndrome [64]. Notably, most cancer patients are born with an intact DNA damage pathway, and the checkpoint is often activated during early stages of cancer progression as an anti-cancer barrier [65-67]. Thus, to reveal how the DNA damage checkpoint fails in cancer cells to prevent tumorigenesis will yield general understanding of cancer progression. In principle, there are two ways by which cancer cells may escape the damage response during cancer progression and therapy: through genetic mutations that cripple the checkpoint pathway, or by hijacking cellular mechanisms that would naturally deactivate the checkpoint. Thus, it is not surprising that known factors of the checkpoint recovery pathway, including Plk1, Aurora A and Wip1, were found to be upregulated in cancers in correlation with more aggressiveness and poor prognosis. The oncogenic potential of these factors was also strongly supported by functional studies in established cancer cell lines and mouse models (reviewed in [3,12,28,68,69]). The role of mitotic kinases in checkpoint recovery further suggests that upregulation of these kinases in cancer cells not only directly promotes cell cycle progression, but also allows cells to bypass the checkpoint-mediated tumor suppression (Figure 3). This knowledge sheds new lights on the oncogenic nature of these kinases, and is of potential value for cancer prevention and diagnosis.

Checkpoint recovery allows cell survival and continued proliferation after DNA damage, and could therefore yield great implications to cancer therapy, especially radiotherapy and chemotherapy using DNA damaging agents. These forms of cancer treatments exploit the toxicity of DNA damage to eliminate tumor cells. Importantly, DNA repair and checkpoint recovery attenuate the killing effect of the treatment and lead to undesired outcomes, and upregulation of the checkpoint recovery mechanism in cancer cells may cause chemoresistance and tumor recurrence (Figure 3). Excitingly, clinical and pre-clinical studies have suggested Plk1, Aurora A and Wip1 as promising drug targets to confer enhanced cancer therapy, consistent with the role of these kinases in checkpoint recovery, mitosis, and other processes. There are currently more than a dozen chemical inhibitors of these factors that are being evaluated in cancer clinical trials [3,12,28]. Future delineation of checkpoint recovery, especially the role of mitotic kinases as essential regulators, will be a key to understanding how tumor cells escape radio- or chemotherapy and relapse, thus building the foundation to enhance cancer therapy.

## Conclusion

A critical aspect of the cellular response to DNA damage is to halt cell cycle progression through the checkpoint mechanism. It has been well-established that activation of the DNA damage checkpoint at G2 leads to inhibition of Cdk1 kinase, the principal mitotic kinase. Interestingly, recent studies in various systems have made a convincing case that Cdk1 is not the only mitotic kinase targeted by the DNA damage checkpoint. Other mitotic kinases, including Plk1, Aurora A, and Gwl, have also been shown to be inhibited by DNA damage as necessary mechanisms to activate the checkpoint and prevent mitotic entry. These lines of evidence thus suggest a “multi-brake” model of checkpoint activation. Conversely, as shown in many recent studies, these mitotic kinases also antagonize checkpoint signaling, and thereby promote checkpoint deactivation and cell cycle reentry, a process termed “checkpoint recovery”. Importantly, the checkpoint recovery process is tightly associated with both cancer progression and treatment. Upregulation of factors involved in checkpoint recovery has been commonly detected in cancer cells, in correlation with aggressive cancer progression and poor treatment outcome. Further delineation of the mutual relationship between mitotic kinases and the DNA damage checkpoint may yield valuable information to improve cancer prevention, diagnosis and treatment in the near future.

## Abbreviations

Cdk: Cyclin-dependent kinase; Plk1: Polo-like kinase 1; Gwl: Greatwall; ATM: Ataxia telangiectasia mutated; ATR: Ataxia telangiectasia and Rad3-related; Wip1: Wild-type p53-induced phosphatase 1; PP2A: Protein phosphatase 2A; Brca1: Breast cancer susceptibility gene 1; 53BP1: p53-binding protein 1; Mdc1: Mediator of DNA damage checkpoint protein 1; FoxM1: Forkhead box protein M1.

## Competing interests

The author declares no competing interest.
